# Dynamometric Strength Profile of Hip Muscles in Youth Soccer Players

**DOI:** 10.3390/ijerph20021291

**Published:** 2023-01-11

**Authors:** Guido Contreras-Díaz, Luis Javier Chirosa-Ríos, Ignacio Chirosa-Ríos, Antonio Riego-Ruiz, Leonardo Intelangelo, Marcelo Tuesta-Roa, Jorge Morales-Zúñiga, Daniel Jerez-Mayorga

**Affiliations:** 1 Department Physical Education and Sports, Faculty of Sport Science, University of Granada, 18011 Granada, Spain; 2 Department of Health, University of Los Lagos, Puerto Montt 5500000, Chile; 3 Musculoskeletal Research Group, University Center for Assistance, Teaching and Research, University of Gran Rosario, Rosario S2000, Argentina; 4 Exercise and Rehabilitation Sciences Laboratory, School of Physical Therapy, Faculty of Rehabilitation Science, University Andres Bello, Santiago 7591538, Chile; 5 Laboratory of Sport Sciences, Center of Medicine Sports MD, Viña del Mar 2521156, Chile

**Keywords:** muscle strength, dynamometer, hip joint, soccer players

## Abstract

Background: Soccer is the most widely practiced sport in the world, demanding high-speed activities such as jumps, sprints and changes of direction. Therefore, having optimal levels of muscle strength improves performance and reduces the injury rate. Objectives: The objectives of our study were (i) to determine the dynamometric profile of hip muscle strength in young soccer players by position, evaluated at different isokinetic speeds, (ii) to describe the conventional and functional unilateral muscle strength ratios, (iii) to analyze the bilateral balance. Methods: Thirty-seven male soccer players (age 17.02 ± 0.92 years) participated in the study. Strength assessment was performed with a functional electromechanical dynamometer, and concentric and eccentric strength of abductors, adductors, extensors and hip flexors were measured bilaterally at 0.5 m/s and 1 m/s. Results: For eccentric right hip abduction at 0.5 m/s, defenders are significantly stronger than midfielders (*p* = 0.013) and stronger than forwards (*p* = 0.140). For eccentric right hip adduction at 0.5 m/s, defenders are significantly stronger than midfielders (*p* = 0.005) and stronger than forwards (*p* = 0.253), as for eccentric right hip adduction at 1 m/s, defenders are significantly stronger than midfielders (*p* = 0.014) and stronger than forwards (*p* = 0.084). There is a significant effect for the conventional strength ratio of left abduction/adduction at 1 m/s. The conventional strength ratio of forwards is significantly higher than that of defenders (*p* = 0.045) and higher than that of midfielders (*p* = 0.152). Conclusions: Concentric and eccentric hip strength values differ according to playing position.

## 1. Introduction

Soccer is the most widely practiced sport in the world, and its performance is determined by technical, tactical, physiological, biomechanical, and psychological factors [1]. For 90 min, the soccer player must perform activities at high speed, including jumps, sprinting, and changes of direction [1]. Therefore, having optimal levels of muscle strength improves performance [2,3] and reduces the injury rate [4,5,6]. Likewise, knowing the muscle strength levels of the player establishes reference values per player and per position [7], which are important to generate guidelines for prevention, rehabilitation, and sports training [8].

Most existing studies on muscle strength parameters and profiles by position in soccer players have been developed at the knee level, evaluating the concentric and eccentric action of quadriceps and hamstrings [9,10,11]. However, it is also necessary to know these values at the hip level due to the great influence they have on sports performance [12,13,14,15] and lower extremity injuries [16]. There is scarce evidence regarding hip muscle strength values in soccer players by position. A recent study found no significant differences between goalkeepers, defenders, midfielders and forwards [17].

In addition to muscle strength, it is important to know the conventional and functional unilateral strength ratios, which are well described in the knee [18], and to a lesser extent, in the hip, focusing mainly on abductors and adductors [19,20], with no further information on flexors and extensors. Another important factor is bilateral strength balance, which is also well described in the knee [21], but in the hip, information is scarce, again emphasizing abductors and adductors [20].

There are several methods and evaluation instruments to determine the levels of muscle strength of the athlete. Manual tests, manual dynamometry, and isokinetic dynamometry are the most frequently used [22]. Isokinetic dynamometry is considered the gold standard for the assessment of muscle function [23]. Though its lack of functionality is questioned, arguing that joints do not function in isolation in sports, this criticism may be less relevant during muscle assessment since its main objective is to find bilateral differences, determine strength ratios, and compare its results with established reference values [23]. In turn, there are valid multi-joint dynamometers, such as the functional electromechanical dynamometer (FEMD) [24], that allow a reliable isokinetic assessment [25,26], respecting the athlete’s natural movement, which can be replicated during evaluation, training, and rehabilitation.

The objectives of our study were (i) to determine the dynamometric profile of hip muscle strength in young soccer players by position, evaluated at different isokinetic speeds, (ii) to describe the conventional and functional unilateral muscle strength ratios, (iii) to analyze the bilateral balance of young soccer players by position on the field.

## 2. Materials and Methods

### 2.1. Study Design

A cross-sectional study design was used. All soccer players and coaching staff were informed about the risks and benefits of the study. Before the evaluations, informed consent was collected from all players. The study was approved by the scientific ethics committee of the University of Los Lagos, Puerto Montt, Chile (N°H007/2022). The research was carried out following the Helsinki declaration’s ethical norms and the sports sciences’ ethical norms [27].

### 2.2. Participants

The study included 40 male soccer players (age 17.02 ± 0.92 years, height 1.70 ± 0.04 cm, weight 66.75 ± 6.07 kg, BMI 22.82 ± 1.57 kg/m^2^), all of them members of the youth team of the professional soccer club of the city of Puerto Montt, Chile, which currently participates in the second division of the national championship, known as first b. To maintain the homogeneity of the subjects, the three goalkeepers of the team were excluded from the study (Table 1). The experience of the players averaged 5 years in the club, and the evaluations were carried out during the first week of preseason. After the evaluations and before the start of the southern zone championship, the players prepared 5 days a week for one month.

### 2.3. Anthropometric Measurement

Body composition was established by measuring weight and height using a digital scale (SECA 769) for weight and a portable stadiometer (SECA 206^®^; Hamburg, Germany) for height.

### 2.4. Muscle Strength

Concentric (CON) and eccentric (ECC) muscle strength of the hip muscles was assessed with a FEMD (Haefni Health System 1.0) [24]. The movements evaluated were hip abduction (ABD), adduction (ADD), extension (EXT) and flexion (FLE). Each movement was evaluated at two speeds (0.5 m/s and 1 m/s) bilaterally. Maximal and relative strength values were recorded. Conventional unilateral strength ratios and functional and bilateral strength balance of the stronger leg with respect to the weaker leg were calculated [28].

(1) $$\mathrm{Bilateral}\;\mathrm{Strength}\;\mathrm{Balance}\;\left(\%\right)=\frac{\mathrm{Strong}\;\mathrm{Leg}\;-\;\mathrm{Weak}\;\mathrm{Leg}}{\mathrm{Strong}\;\mathrm{Leg}}\times 100$$

### 2.5. Experimental Procedure

**Figure 1:**
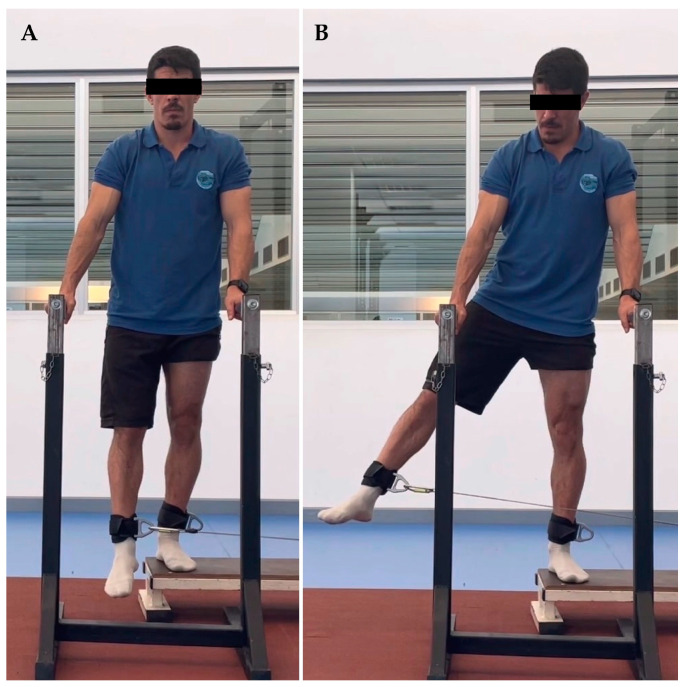
Hip abduction. (**A**) initial position, (**B**) final position.

**Figure 2:**
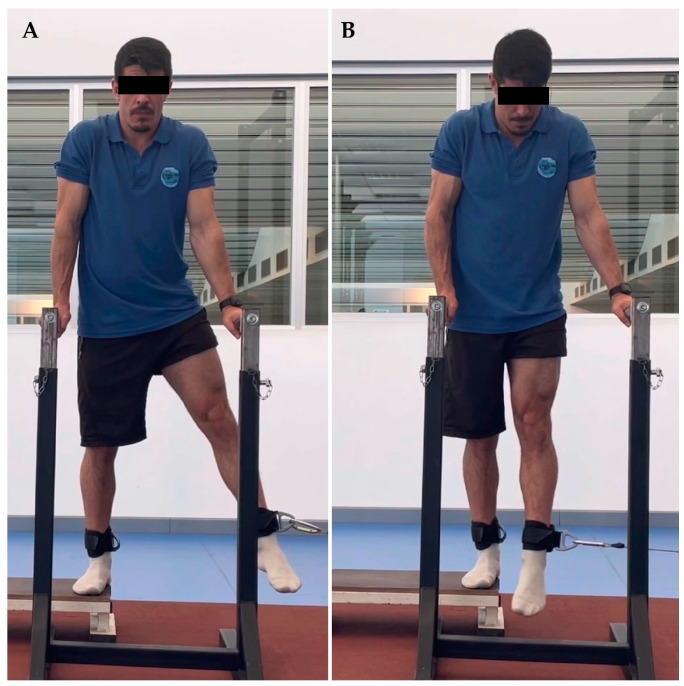
Hip adduction. (**A**) initial position, (**B**) final position.

**Figure 3:**
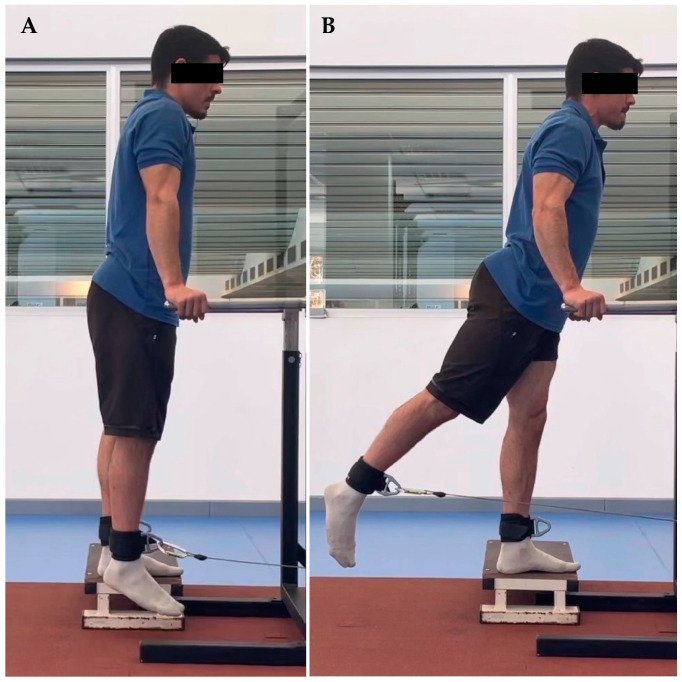
Hip extension. (**A**) initial position, (**B**) final position.

**Figure 4:**
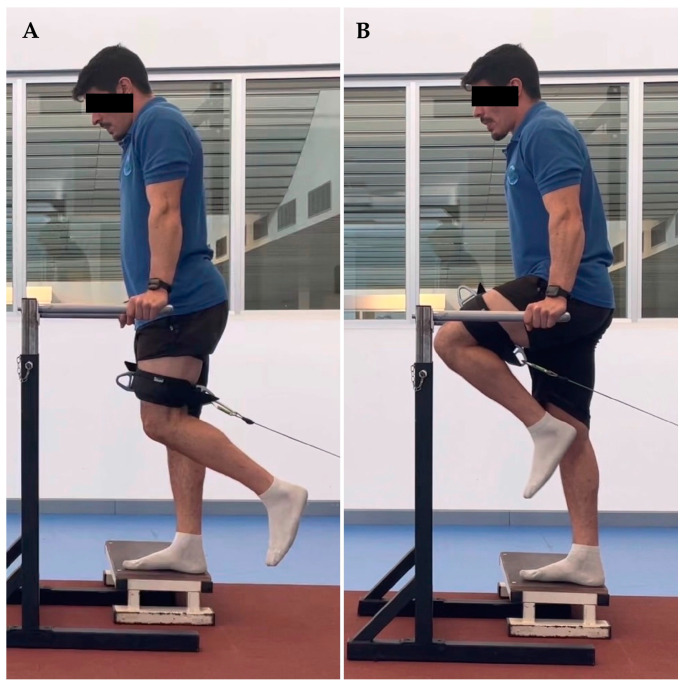
Hip flexion. (**A**) initial position, (**B**) final position.

The thirty-seven youth soccer players were evaluated at the Kinesiology laboratory of the University of Los Lagos, Puerto Montt. Before starting the strength evaluation, the players completed a sports form which included personal, medical, sports and injury history from the previous two years. Hip range of motion was determined with a goniometer, allowing a functional range for each player. The range of abduction was between 10 and 30°, adduction between 30 and 0°, extension from 0 to 30°, and flexion between 0 and 90°. Subsequently, the players performed a 10-min warm-up jogging at 8 km/h., and were familiarized with all hip movements in both limbs at 0.5 m/s. According to previous studies’ results [29,30], the assessments were performed in a standing position. For each movement and speed, two sets of five repetitions were performed with thirty seconds of rest between sets. The order of strength evaluation was (1) left abduction at 0.5 and 1 m/s, (2) right abduction at 0.5 and 1 m/s, (3) left adduction at 0.5 and 1 m/s, (4) right adduction at 0.5 and 1 m/s, (5) left extension at 0.5 and 1 m/s, (6) right extension at 0.5 and 1 m/s, (7) left flexion at 0.5 and 1 m/s and (8) right flexion at 0.5 and 1 m/s (Figure 1, Figure 2, Figure 3 and Figure 4).

All measurements were performed by the same researcher, following the same protocol for each player (Supplementary Material, Video S1).

### 2.6. Statistical Analysis

Descriptive data are presented as mean and standard deviation (SD). The normal distribution of the data (Shapiro-Wilk test) and the homogeneity of variances (Levene test) were confirmed (*p* > 0.05). For the main analysis, analysis of variance (ANOVA) was conducted with Tukey Post-hoc analysis. The Greenhouse–Geisser correction was used when the Mauchly sphericity test was violated. Omega squared (ω^2^) was calculated for the ANOVA where the values of the effect size 0.01, 0.06 and above 0.14 were considered small, medium, and large, respectively [31]. Statistical significance was accepted at *p* < 0.05 level. The JASP statistics package (version 0.16.4) was used for statistical analyses.

## 3. Results

### 3.1. Absolute (N · m) and Relative (N · m/kg) Muscular Strength

There is a significant effect for absolute muscle strength of right hip ECC abduction at 0.5 m/s (F _(2, 34)_ = 4.707, *p* = 0.016, ES = 0.167). Post Hoc analysis using Tukey’s correction revealed that for ECC, abduction defenders are significantly stronger than midfielders (*p* = 0.013) and stronger than forwards (*p* = 0.140) (Table 2). The multifactorial ANOVA showed no significant effect for the other values of absolute and relative hip abduction muscle strength.

There is a significant effect for right hip ECC adduction absolute muscle strength at 0.5 m/s (F _(2, 34)_ = 5.770, *p* = 0.007, ES = 0.205), and for right hip ECC adduction absolute muscle strength at 1 m/s (F _(2, 34)_ = 4.842, *p* = 0.014, ES = 0.172). Post hoc analysis using Tukey’s correction revealed that for right hip ECC adduction at 0.5 m/s, defenders are significantly stronger than midfielders (*p* = 0.005) and stronger than forwards (*p* = 0.253), as for right hip ECC adduction absolute strength at 1 m/s, where defenders are significantly stronger than midfielders (*p* = 0.014) and stronger than forwards (*p* = 0.084) (Table 2). The multifactorial ANOVA showed no significant effect for the other values of absolute and relative hip adduction muscle strength.

Multifactorial ANOVA showed no significant effect on absolute and relative muscle strength values of left and right hip extension and flexion CON and ECC at 0.5 m/s and 1 m/s among defenders, midfielders, and forwards.

### 3.2. Unilateral Conventional Strength Ratio

There is a significant effect for the conventional strength ratio CON/CON of left ABD/ADD at 1 m/s (F _(2, 34)_ = 3.389, *p* = 0.045, ES = 0.114). Post hoc analysis using Tukey’s correction revealed that the conventional strength ratio of forwards is significantly greater than defenders (*p* = 0.045) and greater than midfielders (*p* = 0.152) (Table 3). Multifactorial ANOVA showed no significant effect on the conventional strength ratio CON/CON and ECC/ECC of ABD/ADD between left and right at 0.5 m/s, and FLE/EXT left and right at 0.5 m/s and 1 m/s (Table 3).

### 3.3. Unilateral Functional Strength Ratio

Multifactorial ANOVA showed no significant effect on the functional strength ratio CON/ECC and ECC/CON of ABD/ADD and left and right FLE/EXT at 0.5 m/s and 1 m/s (Table 3).

### 3.4. Bilateral Strength Balance

Multifactorial ANOVA showed no significant effect on bilateral CON and ECC strength balance for hip abduction, adduction, hip extension and hip flexion at 0.5 m/s and 1 m/s among defenders, midfielders, and forwards (Table 4).

## 4. Discussion

The aim of the present study was to determine a dynamometric profile of hip muscle strength in young soccer players according to the position they use in the field (defenders, midfielders, forwards). The main results show that for right ECC abduction at 0.5 m/s and right ECC adduction at 0.5 and 1 m/s, defenders are significantly stronger than midfielders and stronger than forwards. On the other hand, there is no significant difference in hip extension and flexion between defenders, midfielders, and forwards. With respect to the conventional strength ratio, there is a significant difference in the CON/CON ratio of left ABD/ADD at 1 m/s, with the strength ratio of forwards being significantly higher with respect to defenders and higher in forwards with respect to midfielders. For the ABD/ADD and FLE/EXT functional strength ratio, there is no significant effect between defenders, midfielders, and forwards, as well as for bilateral strength balance.

The main conclusions of this study were that eccentric hip abduction and adduction muscle strength was greater in defenders, conventional ABD/ADD strength ratio was greater in forwards, and there is no difference between defenders, midfielders, and forwards with respect to bilateral strength balance.

### 4.1. Absolute and Relative Maximum Strength of Hip Abduction and Adduction

We observed a significant effect for ECC right abduction at 0.5 m/s, demonstrating that defenders are stronger than both midfielders and forwards. These results coincide with the findings found by Wik et al. [11], showing that defenders showed higher eccentric abduction strength values than goalkeepers, with a strong effect size (*p* < 0.05, d = 0.85–0.87). However, midfielders turned out to have higher eccentric hip abduction strength than archers (dominant *p* < 0.01, d = 1.0; non-dominant *p* < 0.05, d = 0.70) and forwards (hip dominant *p* < 0.05, d = 0.54). A significant effect is also observed for ECC right adduction at 0.5 m/s and 1 m/s showing that defenders are stronger than midfielders and forwards, unlike the results of Wik et al. [11], who conclude that midfielders have stronger ECC adduction values with respect to goalkeepers (*p* < 0.05, d = 0.65–0.70) and forwards (*p* < 0.05, d = 0.52–0.57). It is important to mention that Wik et al. [11] used a hand-held dynamometer and a side-lying position to assess eccentric hip strength.

As in our study, Karatrantou et al. [19] found that the peak torque values during eccentric muscle action were significantly (*p* < 0.001) higher compared to those observed during concentric muscle action. This may be due to the fact that during cutting movements or changes of direction, there is great participation of the hip muscles, both in braking and propulsion [7]; likewise, during the ball strike, there is eccentric participation of the hip adductors [8]. Similarly to Wik et al. [11], Karantrantou et al. [19] used the side position to evaluate hip strength; however, the instrument used was an isokinetic dynamometer.

### 4.2. Absolute and Relative Maximum Strength of Hip Flexion and Extension

Our results show that there is no significant difference in the absolute and relative muscle strength values for hip extension and flexion, which agree with the findings of AlTaweel et al. [17], who found no significant differences (*p* > 0.05) between goalkeepers, defenders, midfielders, and forwards. It is important to consider that the position used for the evaluation of hip flexors and extensors used by AlTaweel et al. [17] was supine, and the instrument used was an isokinetic dynamometer. More studies comparing flexor and extensor strength in soccer players by position are needed.

### 4.3. Conventional Strength Ratio

We found that the conventional CON/CON ratio values of ABD/ADD are higher at low velocities (0.5 m/s), unlike the findings of Karatrantou et al. [19], who show that the conventional CON/CON ratio values of ABD/ADD are higher at high velocities (90°/s) (*p* < 0.05). On the other hand, our values of the conventional ECC/ECC ratio of ABD/ADD are higher at high velocities (1 m/s), agreeing with the findings found by Karatrantou et al. [19], who show that the values of conventional ECC/ECC ratio of ABD/ADD are higher at high velocities (90°/s) (*p* < 0.05).

In comparisons of the conventional CON/CON vs. ECC/ECC strength ratio between ABD/ADD at 0.5 m/s and ABD/ADD at 1 m/s, at 0.5 m/s, the conventional CON/CON ratio is higher than the conventional ECC/ECC ratio, however, at 1 m/s the ECC/ECC ratio was higher. These results are related to the types of strength applied in soccer, such as the internal pass, which is executed at high speeds and with a predominance of the eccentric muscle action of adductors [32], and practices associated with displacements, accelerations and changes of direction that increase the eccentric strength of the abductors [33].

For the conventional strength ratio of FLE/EXT, the CON/CON ratio was greater than the ECC/ECC ratio at both speeds. This is perhaps because, for movements in the sagittal plane such as linear sprint, and vertical and horizontal jump, the athlete initiates the movements with a fast concentric action of extensors and then flexors [34,35], and the eccentric phase is mainly determined by muscles surrounding the knee [35].

### 4.4. Functional Strength Ratio

Our research shows that the ABD/ADD CON/ECC functional strength ratio is higher at low velocity (0.5 m/s), and the ECC/CON functional strength ratio is higher at high velocity (1 m/s). These data agree with those reported by Karatrantou et al. [19], who observed in their study that the CON/ECC ratio was higher at low velocity (30°/s), and the ECC/CON ratio increased with increasing angular velocity (90°/s).

For the functional strength ratio of FLE/EXT, the CON/ECC ratio is higher at low velocity (0.5 m/s), and the ECC/CON ratio is higher at high velocity (1 m/s). There are no reports of the functional ratio of hip flexors and extensors.

Comparisons of the functional strength ratio CON/ECC vs. ECC/CON of ABD/ADD and FLE/EXT show that the ECC/CON ratio is always higher, independent of velocity, because when the angular velocity of the movement increases, the maximum strength generation capacity of the antagonist musculature increases through an eccentric action, while the agonist’s muscles produce strength through a concentric action Karatrantou et al. [19].

### 4.5. Bilateral Strength Balance

It is known that in soccer, there are muscle strength imbalances between the extremities, which are justified by age, dominance, and level of training [36]. The literature has pointed out critical values, classifying players as asymmetrical if they have a bilateral strength deficit higher than 15% [37], noting that even deficits over 10% [38] constitute a risk factor for injury. Our results do not show a significant effect between defenders, midfielders, and forwards. However, despite no statistically significant differences, we found many values that are above 15% bilateral CON and ECC deficits at 0.5 m/s and 1 m/s. On the other hand, Helme et al. [39] conclude that functional asymmetry of the lower extremities as a risk factor for injury is moderate to low due to the methodological approach of the studies.

### 4.6. Limitations

This study is not without limitations, as the subjects only had one familiarization session with the FEMD. Therefore, this could have influenced the technical execution of the exercise and, therefore, the peak values of strength. In this context, subjects, being an average age of 17 years old, are just starting strength training, so their practical experience with this type of evaluation is scarce.

## 5. Conclusions

In conclusion, we present a complete profile of hip muscle strength in young soccer players, which may be of interest to coaches, physical trainers, physicians, and physiotherapists, who want to participate in injury prevention, training, rehabilitation, and sports reintegration programs from an early age. There are significant differences in the absolute strength of eccentric hip abduction and adduction, with defenders having greater strength levels. However, we did not find significant differences in the absolute and relative strength of hip flexors and extensors. In the conventional unilateral strength ratio, we found that the forwards have a higher concentric abductor/adductor strength ratio, unlike the functional unilateral strength ratio, where there are no significant differences. These differences may be related to the function of the soccer player within the field since each position demands different capacities. Finally, we also found no significant differences in bilateral strength balance between defenders, midfielders, and forwards.

## Figures and Tables

**Table 1:** Physical characteristics of the participants.

Variable	Defenders (*n* = 13) Mean ± SD	Midfielders (*n* = 12) Mean ± SD	Forwards (*n* = 12) Mean ± SD
Age (years)	17.07 ± 0.49	16.75 ± 1.35	17.25 ± 0.75
Weight (kg)	70.23 ± 5.06	63.16 ± 6.67	66.58 ± 4.46
Height (m)	1.74 ± 0.04	1.69 ± 0.04	1.68 ± 0.04
BMI (kg/m^2^)	23.11 ± 1.26	21.84 ± 1.75	23.50 ± 1.27

BMI: body mass index, SD: standard deviation.

**Table 2:** Absolute (N · m) and relative (N · m/kg) left and right hip strength at 0.5 m/s and 1 m/s.

Side	Velocity	Movement	Maximum Strength	Defenders (*n* = 13) Mean ± SD	Midfielders (*n* = 12) Mean ± SD	Forwards (*n* = 12) Mean ± SD	*p*-Value	ES
Left	0.5 m/seg	ABD CON	Absolute	256.00 ± 101.52	201.08 ± 37.62	238.25 ± 46.47	0.147	0.052
			Relative	3.62 ± 1.35	3.22 ± 0.74	3.60 ± 0.86	0.569	0.000
		ABD ECC	Absolute	310.61 ± 47.74	275.25 ± 46.36	317.50 ± 59.55	0.111	0.000
			Relative	4.43 ± 0.70	4.38 ± 0.78	4.81 ± 1.05	0.421	0.000
		ADD CON	Absolute	287.84 ± 67.64	252.41 ± 80.24	266.50 ± 28.67	0.375	0.000
			Relative	4.08 ± 0.87	3.98 ± 1.10	4.02 ± 0.54	0.955	0.000
		ADD ECC	Absolute	404.38 ± 82.59	351.25 ± 74.97	381.33 ± 57.17	0.203	0.035
			Relative	5.74 ± 1.00	5.61 ± 1.34	5.77 ± 1.10	0.939	0.000
		EXT CON	Absolute	307.15 ± 74.92	289.00 ± 85.33	304.16 ± 37.97	0.786	0.000
			Relative	4.35 ± 0.90	4.58 ± 1.26	4.60 ± 0.81	0.779	0.000
		EXT ECC	Absolute	416.53 ± 84.03	395.16 ± 98.72	436.75 ± 113.49	0.594	0.000
			Relative	5.91 ± 0.98	6.33 ± 1.79	6.59 ± 1.78	0.549	0.000
		FLE CON	Absolute	464.15 ± 136.11	406.58 ± 95.32	430.33 ± 106.35	0.458	0.000
			Relative	6.60 ± 1.90	6.48 ± 1.52	6.48 ± 1.60	0.979	0.000
		FLE ECC	Absolute	567.15 ± 86.27	518.91 ± 70.82	538.08 ± 63.85	0.278	0.017
			Relative	8.10 ± 1.27	8.30 ± 1.45	8.13 ± 1.25	0.924	0.000
Right	0.5 m/seg	ABD CON	Absolute	235.69 ± 54.90	192.83 ± 41.16	227.08 ± 46.36	0.079	0.085
			Relative	3.36 ± 0.82	3.06 ± 0.59	3.42 ± 0.73	0.425	0.000
		ABD ECC	Absolute	333.07 ± 77.38	257.41 ± 54.64	284.08 ± 51.35	0.016 *	0.167
			Relative	4.78 ± 1.28	4.11 ± 0.93	4.32 ± 1.08	0.312	0.010
		ADD CON	Absolute	292.69 ± 72.18	256.08 ± 46.58	277.00 ± 30.46	0.242	0.025
			Relative	4.15 ± 0.93	4.06 ± 0.63	4.18 ± 0.62	0.913	0.000
		ADD ECC	Absolute	420.38 ± 86.30	324.41 ± 62.77	374.75 ± 57.68	0.007 *	0.205
			Relative	6.00 ± 1.26	5.13 ± 0.75	5.66 ± 1.00	0.125	0.061
		EXT CON	Absolute	331.07 ± 80.05	301.41 ± 67.67	335.33 ± 61.54	0.444	0.000
			Relative	4.70 ± 1.02	4.78 ± 0.97	5.06 ± 1.00	0.651	0.000
		EXT ECC	Absolute	396.84 ± 53.13	383.58 ± 80.89	406.16 ± 72.98	0.728	0.000
			Relative	5.65 ± 0.63	6.15 ± 1.58	6.13 ± 1.28	0.511	0.000
		FLE CON	Absolute	487.61 ± 106.33	396.66 ± 107.26	448.33 ± 130.54	0.156	0.049
			Relative	6.92 ± 1.33	6.37 ± 1.94	6.75 ± 1.98	0.729	0.000
		FLE ECC	Absolute	586.38 ± 83.96	511.50 ± 85.82	540.75 ± 101.22	0.128	0.060
			Relative	8.35 ± 1.12	8.11 ± 1.27	8.16 ± 1.68	0.899	0.000
Left	1 m/seg	ABD CON	Absolute	218.69 ± 71.89	207.58 ± 69.12	268.33 ± 103.66	0.173	0.043
			Relative	3.12 ± 1.07	3.34 ± 1.25	4.06 ± 1.72	0.216	0.031
		ABD ECC	Absolute	416.07 ± 149.60	357.50 ± 94.60	381.66 ± 117.90	0.498	0.000
			Relative	5.95 ± 2.19	5.69 ± 1.62	5.80 ± 2.01	0.948	0.000
		ADD CON	Absolute	306.30 ± 85.01	266.33 ± 90.39	265.25 ± 66.50	0.362	0.002
			Relative	4.33 ± 1.08	4.20 ± 1.32	4.02 ± 1.15	0.803	0.000
		ADD ECC	Absolute	435.07 ± 72.14	379.25 ± 53.26	401.16 ± 50.81	0.077	0.087
			Relative	6.18 ± 0.90	5.99 ± 0.48	6.07 ± 1.04	0.849	0.000
		EXT CON	Absolute	316.92 ± 77.74	296.33 ± 87.19	295.66 ± 53.91	0.718	0.000
			Relative	4.48 ± 0.85	4.71 ± 1.32	4.46 ± 0.91	0.810	0.000
		EXT ECC	Absolute	557.84 ± 177.82	535.91 ± 187.89	509.33 ± 169.37	0.795	0.000
			Relative	7.86 ± 2.08	8.49 ± 2.93	7.69 ± 2.60	0.718	0.000
		FLE CON	Absolute	436.84 ± 112.86	412.58 ± 122.75	393.41 ± 96.41	0.623	0.000
			Relative	6.17 ± 1.26	6.60 ± 2.10	5.92 ± 1.49	0.600	0.000
		FLE ECC	Absolute	668.46 ± 79.06	622.16 ± 71.08	611.16 ± 81.21	0.157	0.049
			Relative	9.55 ± 1.23	9.92 ± 1.38	9.26 ± 1.77	0.555	0.000
Right	1 m/seg	ABD CON	Absolute	209.76 ± 41.51	187.16 ± 33.39	210.58 ± 49.69	0.309	0.011
			Relative	2.98 ± 0.55	2.97 ± 0.52	3.19 ± 0.90	0.678	0.000
		ABD ECC	Absolute	413.07 ± 119.00	360.16 ± 94.46	357.58 ± 88.32	0.316	0.010
			Relative	5.93 ± 1.86	5.74 ± 1.61	5.44 ± 1.63	0.778	0.000
		ADD CON	Absolute	290.30 ± 64.10	295.91 ± 111.53	283.33 ± 56.18	0.929	0.000
			Relative	4.12 ± 0.78	4.71 ± 1.87	4.27 ± 0.89	0.492	0.000
		ADD ECC	Absolute	459.76 ± 93.18	357.91 ± 62.92	384.41 ± 95.08	0.014 *	0.172
			Relative	6.57 ± 1.37	5.66 ± 0.68	5.82 ± 1.54	0.170	0.044
		EXT CON	Absolute	336.07 ± 80.32	319.75 ± 75.55	322.75 ± 47.53	0.822	0.000
			Relative	4.75 ± 0.93	5.08 ± 1.12	4.86 ± 0.75	0.692	0.000
		EXT ECC	Absolute	508.23 ± 122.76	566.41 ± 171.39	520.25 ± 169.96	0.623	0.000
			Relative	7.26 ± 1.79	9.02 ± 2.68	7.90 ± 2.85	0.214	0.031
		FLE CON	Absolute	488.92 ± 125.87	424.83 ± 123.96	430.08 ± 143.53	0.404	0.000
			Relative	6.91 ± 1.53	6.76 ± 1.96	6.49 ± 2.21	0.858	0.000
		FLE ECC	Absolute	674.00 ± 63.71	632.41 ± 113.25	603.16 ± 93.54	0.166	0.045
			Relative	9.60 ± 0.77	9.99 ± 1.30	9.11 ± 1.74	0.277	0.017

ABD: abduction, ADD: adduction, EXT: extension, FLE: flexion, CON: concentric, ECC: eccentric, SD: standard deviation, ES: effect size (Omega squared (ω^2^)), * Significant at 0.05 level.

**Table 3:** Conventional and Functional Unilateral Strength Ratio of Left and Right Hip Abductor/Adductor and Flexor/Extensor at 0.5 m/s and 1 m/s.

Velocity	Unilateral Ratio	Side	Muscle Action	Defenders (*n* = 13) Mean ± SD	Midfielders (*n* = 12) Mean ± SD	Forwards (*n* = 12) Mean ± SD	*p*-Value	ES
0.5 m/seg	ABD/ADD Conventional	Left	CON/CON	89.51 ± 24.85	83.97 ± 18.30	90.45 ± 22.37	0.739	0.000
			ECC/ECC	79.36 ± 18.18	81.40 ± 19.52	85.09 ± 20.10	0.755	0.000
		Right	CON/CON	81.43 ± 11.42	75.46 ± 8.66	81.93 ± 13.70	0.313	0.010
			ECC/ECC	82.30 ± 24.19	81.29 ± 20.40	76.70 ± 14.03	0.765	0.000
1 m/seg	ABD/ADD Conventional	Left	CON/CON	75.140 ± 24.96	81.26 ± 27.80	102.76 ± 30.11	0.045 *	0.114
			ECC/ECC	97.04 ± 33.63	94.52 ± 23.27	95.43 ± 28.19	0.975	0.000
		Right	CON/CON	73.50 ± 12.49	67.91 ± 16.62	76.14 ± 18.76	0.448	0.000
			ECC/ECC	93.14 ± 33.78	101.69 ± 26.22	94.62 ± 16.53	0.701	0.000
0.5 m/seg	ABD/ADD Functional	Left	CON/ECC	63.57 ± 20.64	58.63 ± 11.51	63.88 ± 16.33	0.688	0.000
			ECC/CON	115.17 ± 39.24	116.30 ± 30.61	121. 14 ± 29.71	0.897	0.000
		Right	CON/ECC	56.89 ± 12.24	60.25 ± 12.06	61.20 ± 11.94	0.645	0.000
			ECC/CON	120.96 ± 44.50	102.27 ± 20.98	102.64 ± 14.70	0.219	0.030
1 m/seg	ABD/ADD Functional	Left	CON/ECC	50.67 ± 14.43	55.32 ± 18.49	67.83 ± 26.18	0.106	0.069
			ECC/CON	146.95 ± 64.81	150.21 ± 66.07	153.93 ± 66.88	0.965	0.000
		Right	CON/ECC	46.54 ± 9.89	53.37 ± 11.48	58.41 ± 20.34	0.137	0.056
			ECC/CON	150.69 ± 61.46	134.91 ± 55.86	128.94 ± 31.34	0.555	0.000
0.5 m/seg	FLE/EXT Conventional	Left	CON/CON	150.97 ± 24.40	144.77 ± 29.71	141.87 ± 30.94	0.715	0.000
			ECC/ECC	139.52 ± 26.67	138.93 ± 38.32	129.12 ± 30.87	0.672	0.000
		Right	CON/CON	149.36 ± 20.16	136.42 ± 38.27	136.23 ± 43.79	0.565	0.000
			ECC/ECC	148.51 ± 19.06	135.70 ± 20.39	134.70 ± 23.91	0.202	0.035
1 m/seg	FLE/EXT Conventional	Left	CON/CON	139.97 ± 29.16	141.51 ± 31.39	134.61 ± 29.87	0.841	0.000
			ECC/ECC	128.57 ± 32.92	128.26 ± 41.83	129.22 ± 34.12	0.997	0.000
		Right	CON/CON	150.10 ± 47.04	132.00 ± 19.98	131.45 ± 30.27	0.319	0.009
			ECC/ECC	138.05 ± 28.31	119.26 ± 36.90	122.92 ± 26.16	0.278	0.017
0.5 m/seg	FLE/EXT Functional	Left	CON/ECC	112.140 ± 28.44	107.65 ± 29.48	104.28 ± 34.69	0.816	0.000
			ECC/CON	192.96 ± 46.79	190.15 ± 51.23	178.79 ± 25.53	0.688	0.000
		Right	CON/ECC	122.67 ± 20.57	104.78 ± 26.48	113.54 ± 40.91	0.347	0.004
			ECC/CON	186.04 ± 51.24	173.04 ± 26.25	163.02 ± 27.97	0.316	0.010
1 m/seg	FLE/EXT Functional	Left	CON/ECC	81.37 ± 17.48	79.85 ± 19.67	85.36 ± 32.42	0.844	0.000
			ECC/CON	219.65 ± 44.57	224.53 ± 65.90	211.74 ± 39.79	0.826	0.000
		Right	CON/ECC	100.13 ± 31.73	78.80 ± 25.25	89.31 ± 35.35	0.244	0.024
			ECC/CON	209.52 ± 47.16	204.91 ± 49.78	189.04 ± 30.27	0.478	0.000

ABD/ADD: abduction/adduction, FLE/EXT: flexion/extension, CON/ECC: concentric/eccentric, ECC/CON: eccentric/concentric, SD: standard deviation, ES: effect size (Omega squared (ω^2^)). * Significant at 0.05 level.

**Table 4:** Bilateral Concentric and Eccentric Hip Strength Balance (%) at 0.5 m/s and 1 m/s.

Bilateral Strength Balance	Velocity	Muscle Action	Defenders (*n* = 13) Mean ± SD	Midfielders (*n* = 12) Mean ± SD	Forwards (*n* = 12) Mean ± SD	*p*-Value	ES
ABD/ABD	0.5 m/seg	CON	13.53 ± 13.31	11.85 ± 7.61	15.78 ± 13.71	0.722	0.000
		ECC	14.63 ± 10.84	12.62 ± 10.19	12.12 ± 16.20	0.871	0.000
	1 m/seg	CON	13.81 ± 10.59	17.71 ± 16.44	18.82 ± 15.08	0.650	0.000
		ECC	15.49 ± 20.04	11.72 ± 12.47	16.03 ± 11.78	0.756	0.000
ADD/ADD	0.5 m/seg	CON	9.77 ± 9.28	14.47 ± 11.79	10.69 ± 7.29	0.446	0.000
		ECC	10.96 ± 11.99	13.52 ± 13.38	10.66 ± 4.61	0.773	0.000
	1 m/seg	CON	16.01 ± 11.56	16.61 ± 11.93	19.35 ± 12.90	0.769	0.000
		ECC	14.11 ± 11.06	10.41 ± 5.38	13.52 ± 8.17	0.526	0.000
EXT/EXT	0.5 m/seg	CON	8.95 ± 6.57	10.54 ± 5.10	11.04 ± 10.96	0.786	0.000
		ECC	7.11 ± 4.31	12.22 ± 14.23	10.02 ± 12.62	0.519	0.000
	1 m/seg	CON	12.14 ± 6.33	14.27 ± 6.35	13.65 ± 9.31	0.761	0.000
		ECC	17.50 ± 15.22	25.23 ± 17.63	19.58 ± 12.16	0.434	0.000
FLE/FLE	0.5 m/seg	CON	11.80 ± 7.50	16.99 ± 19.31	11.56 ± 7.49	0.493	0.000
		ECC	8.10 ± 5.69	11.24 ± 9.08	9.06 ± 9.18	0.619	0.000
	1 m/seg	CON	16.60 ± 14.95	14.64 ± 9.82	22.03 ± 14.68	0.385	0.000
		ECC	6.11 ± 4.15	12.31 ± 9.92	7.57 ± 5.91	0.087	0.080

ABD/ABD: abduction/abduction, ADD/ADD: adduction/adduction, EXT/EXT: extension/extension, FLE/FLE: flexion/flexion, CON: concentric, ECC: eccentric, ES: effect size (Omega squared (ω^2^)).

## Data Availability

Not applicable.

## Supplementary Materials

The following supporting information can be downloaded at: https://www.mdpi.com/article/10.3390/ijerph20021291/s1, Video S1: Experimental Procedure.
